# Potential cosmeceutical lamellar liquid crystals containing black longan (*Dimocarpus longan* Lour.) seed extract for MMP-1 and hyaluronidase inhibition

**DOI:** 10.1038/s41598-022-11554-5

**Published:** 2022-05-10

**Authors:** Preaploy Hong-in, Wantida Chaiyana

**Affiliations:** 1 Master’s Degree Program in Cosmetic Science, Faculty of Pharmacy, Chiang Mai University, Chiang Mai, 50200 Thailand; 2 Department of Pharmaceutical Sciences, Faculty of Pharmacy, Chiang Mai University, Chiang Mai, 50200 Thailand; 3 Research Center of Pharmaceutical Nanotechnology, Faculty of Pharmacy, Chiang Mai University, Chiang Mai, 50200 Thailand; 4 Innovation Center for Holistic Health, Nutraceuticals, and Cosmeceuticals, Faculty of Pharmacy, Chiang Mai University, Chiang Mai, 50200 Thailand

**Keywords:** Plant sciences, Health care

## Abstract

The aims of this study were to evaluate the biological activities of black *Dimocarpus longan* Lour. seed extracts and develop a lamellar liquid crystal (LLC). Different solvents, including petroleum ether, ethyl acetate, and 95% *v*/*v* ethanol, were used in the maceration of black *D. longan* seeds. The inhibitory activities on matrix metalloproteinase-1 (MMP-1) and hyaluronidase were evaluated. The irritating potency of *D. longan* seed extracts was determined using the hen's egg chorioallantoic membrane test. The extract with the strongest anti-ageing activities and no irritant impact was examined for its chemical contents using high-performance liquid chromatography and incorporated into the LLC. Various factors affecting the LLC formulations, including surfactant types and amounts, thickening agent types and amounts, and various oil types, were investigated. The results demonstrated that the ethyl acetate extract (EtOAc) was the most potent inhibitor of MMP-1 (IC_50_ = 21.7 ± 5.4 µg/mL) and hyaluronidase (oleanolic acid equivalent = 0.44 ± 0.03 g per g extract). Interestingly, its MMP-1 inhibition was equivalent to that of oleanolic acid, corilagin, and gallic acid. Furthermore, its hyaluronidase inhibition was equivalent to that of gallic acid and ellagic acid. Gallic acid, which was the most abundant compound (15.6% ± 0.06% *w*/*w*) was suggested as the compound responsible for the biological activities of EtOAc extract. Considering its potential anti-skin ageing properties with no irritation of EtOAc extract, it was incorporated into the most suitable LLC, which was composed of 5% *w*/*w* Lexfeel® D5 oil, 0.5% *w*/*w* Carbopol® U21, 2% *w*/*w* Liquid Crystal Cream Maker, and 92.5% *w*/*w* DI water. The LLC containing EtOAc extract presented birefringence under a polarizing light microscope, indicating its crystallinity. The formulation had good stability after heating–cooling cycles in terms of external appearance, crystallinity, viscosity, and pH (5.5). As a result, it is recommended as a potential cosmeceutical formulation for anti-skin wrinkling. It is proposed that more research be conducted on the safety and efficacy of the treatment on human volunteers.

## Introduction

The term “cosmeceuticals” is an intentional portmanteau of cosmetics and pharmaceuticals, whereby the products that are used for external skin cleansing and beautification are known as cosmetics^1^, while the products that can prevent, alleviate, treat, or heal disease, as well as influence the structure or function of the physique, are described as pharmaceuticals^2^. In brief, cosmeceuticals are aimed at having an advantageous effect on skin health and beauty, i.e., their properties are involved both cosmetic and therapeutic (medical or drug-like) effects^3^. Numerous biological activities, especially anti-ageing properties, are related to the cosmeceutical effects. Skin ageing is a complex process, accompanied by phenotypic changes in cutaneous cells, as well as structural and functional changes in extracellular matrix (ECM) components, such as collagens, elastin, and proteoglycans that are required to provide tensile strength, elasticity, and hydration to the skin, respectively^4,5^. Therefore, cosmeceuticals which can retard or prevent the ECM degradation would be beneficial for anti-skin ageing.

Black *Dimocarpus longan* Lour. that has undergone a thermal ageing process has been suggested for use as a cosmeceutical active ingredient due to its potent anti‑hyaluronidase and antioxidant activities^6^. The biological active compounds in black *D. longan* fruit, notably the pericarp and seed, have been identified as phenolic compounds^6^. Gallic acid, the most abundant detected, was enhanced tenfold after the thermal and ageing process^6^. Moreover, ellagic acid and corilagin were also detected in various parts of *D. longan* fruit and significantly enhanced after the black *D. longan* production process^6^. These phenolic compounds have been reported for a variety of biological activities related to cosmetic and cosmeceutical applications^7–9^. Since hyaluronidase is an endoglycosidase enzyme capable of hydrolyzing hyaluronic acid, it is a primary catalyst for hyaluronic acid depolymerization and ECM damage^10,11^. On the other hand, free radicals are known as a key factor in the skin ageing process, resulting from a disbalance of oxidant and antioxidant status^12^. The resulting oxidative stress and reactive oxygen species (ROS) lead to the activation of mitogen-activated protein kinase (MAPK), resulting in an increase in the expression of activator protein 1 (AP-1) and matrix metalloproteinases (MMPs), as well as collagen fiber degradation^13^. Since collagen fiber is the major constituent of the skin, accounting for 70%–80% of the total skin weight, a significant factor in skin ageing is collagen degradation by MMP-1^14^. Although black *D. longan* extracts were reported for anti‑hyaluronidase and antioxidant activities in our previous study^6^, their effect on MMP-1, which plays an essential role in the collagen degradation process, has not yet been investigated. Therefore, the present study focused on the inhibitory effects on MMP-1 of black *D. longan* seed extracts, which were previously reported to contain a higher content of biological active compounds and possess more potent biological activities than aril or pericarp extracts.

In addition to a scientifically proven active ingredient, cosmeceuticals must present no deleterious consequences and provide optimum skin penetration^15^. Therefore, black *D. longan* seed extracts were investigated for their irritation properties, and lamellar liquid crystal formulations were developed for delivering the bioactive compounds from black *D. longan* seed extracts into the skin layer. Since lyotropic liquid crystals have been extensively explained in the context of emulsion technology, they can be applied in the pharmaceutical and cosmetic area^16^. In addition to the traditional emulsion system, in which droplets of oil are emulsified by a surfactant and dispersed in water or droplets of water are emulsified by surfactant and dispersed in an oil phase, liquid crystal emulsion is a recent type of emulsion in which the surfactant and oil molecules are in an ordered array at the oil–water interface^17^. Lamellar liquid crystals (LLCs) are also known as layered envelopes of uneven disc-like micelles where the surfactant molecules are attached tail to tail and manipulated in flat layers encountering the water layers^18^. LLCs have presented preferable application performance, including greater effectiveness in the skin whitening effect and higher skin retention of active compounds^18^. The structure of LLCs is arranged similarly to lipids in the cell membrane and intercellular space of the stratum corneum; hence, the enhanced efficacy of LLCs is due to the improved permeability of the active compounds because of the interaction between intercellular lipids in the stratum corneum and LLC emulsifier^18,19^. Moreover, LLCs are different from common emulsions in terms of thermodynamically stability, resulting in higher loading efficacy for the bioactive ingredient and controlled release^20^. However, the formation of LLCs depends on both the preparation procedure and the constituents of the formulation^17^. Therefore, the present study investigated various factors affecting LLC formation, including types and concentration of emulsifiers and oils, as well as the presence of a thickening agent in the formulation. Additionally, LLCs containing black *D. longan* seed extracts, which presented MMP-1-inhibitory activity with no irritation, were developed.

## Results and discussion

### MMP-1-inhibitory activity of black D. longan seed extracts

**Figure 1:**
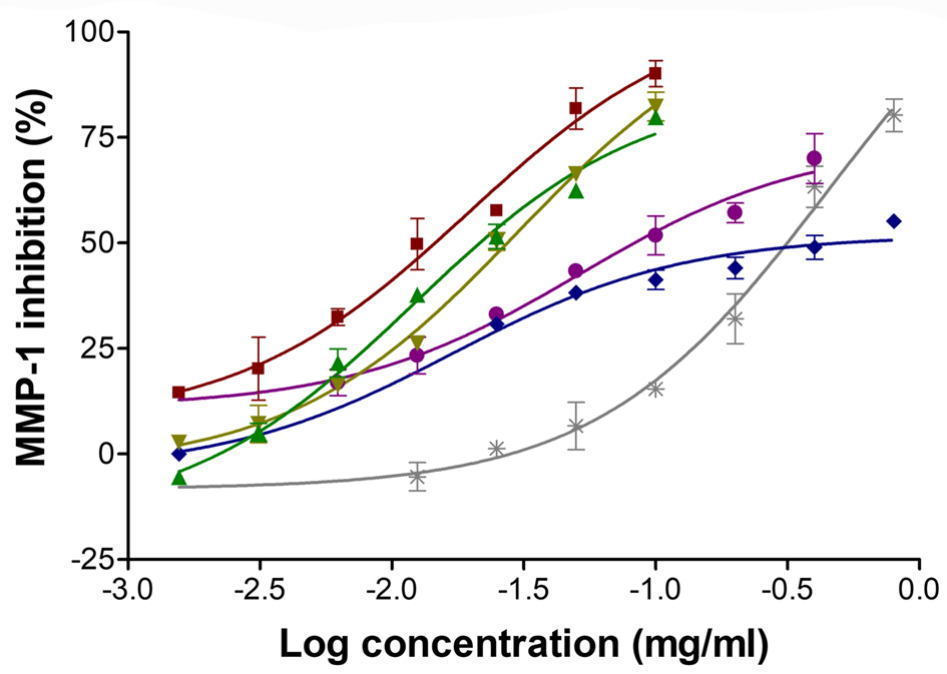
Dose–response curve of black *D. longan* seed extracts, extracted using petroleum ether (blue), ethyl acetate (violet), and 95% *v*/*v* ethanol (gray), indicating matrix metalloproteinase-1 (MMP-1)-inhibitory activity. Oleanolic acid (red), corilagin (green), and gallic acid (yellow) were used as standard compounds.

Three different black *D. longan* seed extracts were obtained from petroleum ether (PET), ethyl acetate (EtOAc), and 95% *v*/*v* ethanol (EtOH), with yields of 1.39%, 1.35%, and 6.60% *w*/*w*, respectively. All extracts were a semisolid mass with dark-brown color and characteristic odor. The MMP-1-inhibitory activity of black *D. longan* seed extracts is shown in Table 1. As demonstrated in Figure 1, all extracts and compounds displayed a dose–response relationship. Among different black *D. longan* seed extracts, EtOAc extract exhibited the most significantly potent MMP-1 inhibition with the lowest IC_50_ value (*p* <0.05), followed by PET and EtOH extract. Interestingly, EtOAc extract was found to be as potent as corilagin, gallic acid, and oleanolic acid. However, ellagic acid, which is a gallic acid derivative, had no effect on the MMP-1 activity. The most plausible explanation could be due to the steric effect of ellagic acid when compared to gallic acid^21^ and the additional functional group that was incongruent with the active site of MMP-1.

**Table 1:** Half-maximal MMP-1 inhibitory concentration (IC_50_) of black *D. longan* seed extracts.

Sample	IC_50_ (µg/ml)
**Positive control**	
Oleanolic acid	12.3 ± 2.9^a^
**Major components**	
Corilagin	20.6 ± 1.1^a^
Gallic acid	30.9 ± 2.8^a,b^
Ellagic acid	N.D
**Black *D. longan* seed extracts**	
PET extract	50.8 ± 5.1^b^
EtOAc extract	21.7 ± 5.4^a^
EtOH extract	453.0 ± 19.6^c^

*N.D*., Not detected; PET extract, black *D. longan* seed petroleum ether extract; EtOAc extract, black *D. longan* seed ethyl acetate extract; EtOH extract, black *D. longan* seed ethanolic extract.

MMPs, which are zinc-dependent endopeptidases that degrade ECM components and play a role in dermal turnover and remodeling, are expressed at very low levels in normal skin and remain inactive due to endogenous inhibitors^22^. However, during the inflammation process, exposure to UV irradiation, and even the natural ageing process, MMPs can be activated, resulting in enhanced ECM breakdown and skin wrinkles^23,24^. Among several types of MMPs, MMP-1 (interstitial collagenase) is the most closely associated with skin wrinkles as it breaks down the extracellular fibers composed of type I and III collagen^25^. Therefore, EtOAc extract can be suggested for further use as a cosmeceutical active ingredient for anti-skin wrinkling since it is a potent MMP-1 inhibitor.

### Hyaluronidase-inhibitory activity of black D. longan seed extracts

**Figure 2:**
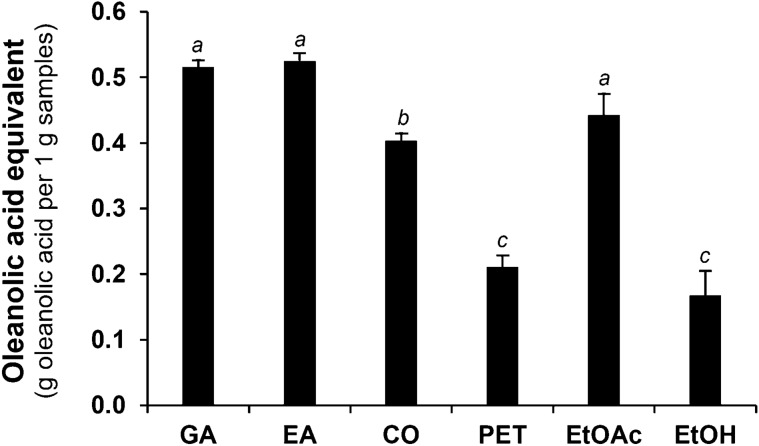
Oleanolic acid equivalent on hyaluronidase inhibition of gallic acid (GA), ellagic acid (EA), corilagin (CO), and black *D. longan* seed extracts, extracted using petroleum ether (PET), ethyl acetate (EtOAc), and 95% *v*/*v* ethanol (EtOH). Different letters, a, b, and c, denote a statistically significant difference in oleanolic acid equivalents among samples (*p* < 0.05).

The hyaluronidase-inhibitory activity of black *D. longan* seed extracts is shown in Figure 2. As oleanolic acid has long been known as a natural cosmeceutical component with various skin beneficial effects, particularly hyaluronidase-inhibitory activity^26^, the results of hyaluronidase inhibition were given in terms of oleanolic acid equivalent (OAE) in the present study. Gallic acid, ellagic acid, and corilagin, reported as the major bioactive components of black *D. longan*, were also investigated for their inhibitory effect on hyaluronidase^10^. Among black *D. longan* seed extracts, EtOAc extract was highlighted as the most potent hyaluronidase inhibitor with an OAE of 0.44 ± 0.03 g of oleanolic acid per 1 g sample (*p* < 0.05). PET and EtOH extract inhibited hyaluronidase in a comparable manner with OAEs of 0.21 ± 0.02 and 0.17 ± 0.04 g of oleanolic acid per 1 g sample, respectively.

Hyaluronidase is an enzyme related to skin wrinkle formation considering its key role in the breakdown of hyaluronic acid or hyaluronan, a major component of the three-dimensional network of skin ECM with crucial physiological functions in maintaining the skin structure and retaining water molecules in the skin^27^. Therefore, the anti-skin ageing effects could be attributed to EtOAc extract, which exerted a potent inhibitory effect on hyaluronidase,.

### Irritation effect of black D. longan seed extracts

**Figure 3:**
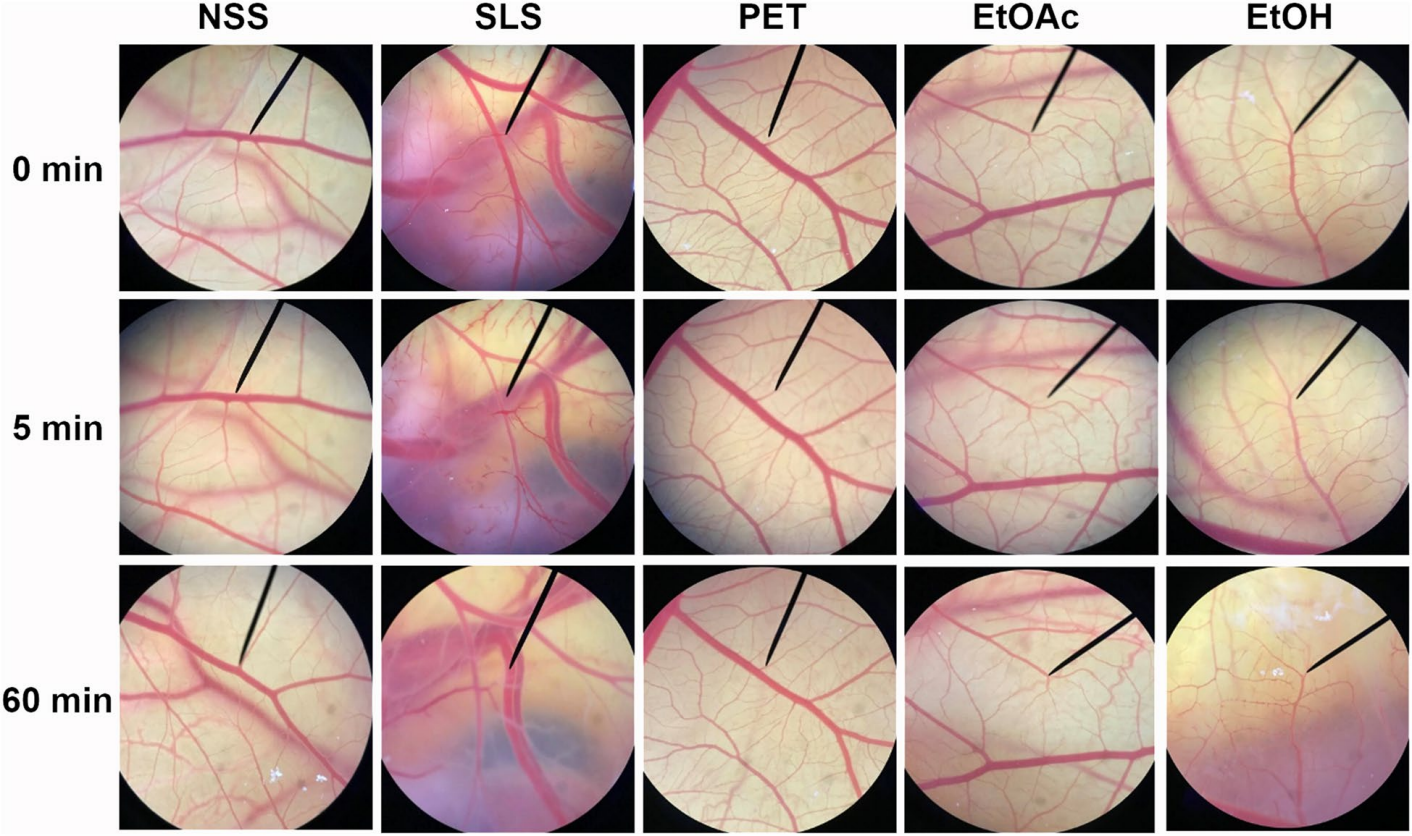
Chorioallantoic membrane of hen’s egg after exposure to normal saline solution (NSS), 1% w/v sodium lauryl sulfate (SLS), black *D. longan* seed petroleum ether extract (PET extract), black *D. longan* seed ethyl acetate extract (EtOAc extract), and black *D. longan* seed ethanolic extract (EtOH extract).

The fertilized hen's egg test on the chorioallantoic membrane (HET-CAM)^28,29^ was used to determine the irritation effect of black *D. longan* seed extracts. This experiment is a well-known and trustworthy irritation study. Because the age of the animal's embryo was less than half of the incubation period, ethical approval was not required. Prior to the experiment, the HET-CAM test was validated using normal saline solution (NSS) as a negative control, whereas sodium lauryl sulfate (SLS) aqueous solution (1% *w*/*v*) was used as a positive control because of its irritating effects on the skin, resulting in increased transepidermal water loss (TEWL) and skin erythema^30^. The results shown in Figure 2 point out that NSS induced no irritation since the CAM showed no such signs, resulting in an irritation score (IS) of 0.0 ± 0.0. In contrast, SLS induced severe irritation with an IS of 10.4 ± 0.8. Hemorrhage, vascular lysis, and vascular coagulation were observed in the CAM after 5 min of exposure to SLS, and further severity can be detected after 1 h. Some small vessels disappeared after 1 h of exposure. Considering the results using NSS and SLS, the HET-CAM test was well validated since the negative control induced no irritation, whereas the positive control induced severe irritation. According to the validated HET-CAM assay, black *D. longan* seed extracts were safe and induced no irritation signs on the CAM after 5 min of exposure, as shown in Figure 3. The IS of all black *D. longan* seed extracts was 0.0 ± 0.0, classified as no irritation. Furthermore, irritation signs were not detected after 1 h of exposure. The results were comparable to those of NSS, the negative control. Since the HET-CAM test has been proposed as a useful in vitro assay for the irritation assessment of cosmetic formulations and ingredients, especially eye irritation^28^, it can be concluded that all black *D. longan* seed extracts are safe for topical application as an active cosmetic ingredient. Because the extracts from black *D. longan* seed, particularly EtOAc extract, demonstrated substantial anti-ageing activities through MMP-1 and hyaluronidase inhibition while producing no irritation, they can be considered safe for further cosmetic or cosmeceutical application.

### Phenolic content of black D. longan seed extracts

**Figure 4:**
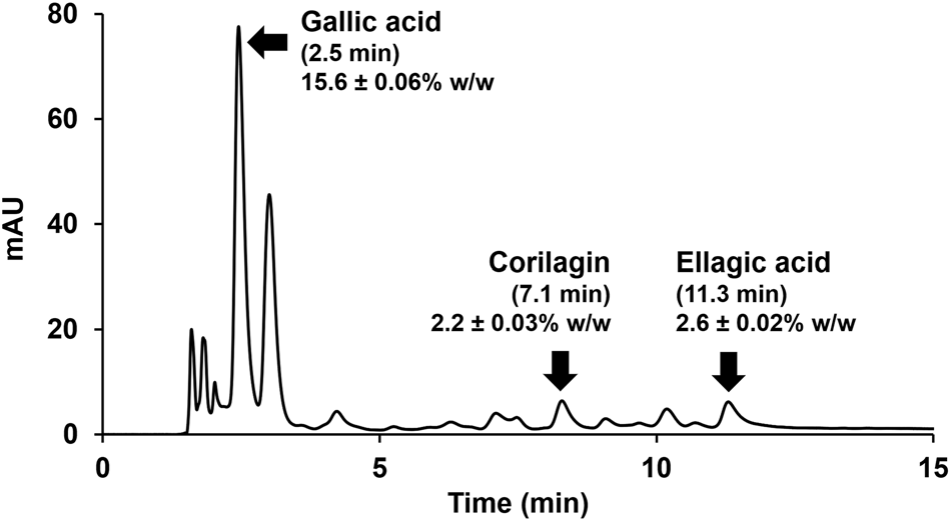
High-performance liquid chromatography (HPLC) chromatograms of black *D. longan* seed ethyl acetate extract (EtOAc).

Regarding EtOAc extract's significant MMP-1- and hyaluronidase-inhibitory effects, it is an appealing natural anti-ageing active component for future cosmetic or cosmeceutical product development. As a result, high-performance liquid chromatography (HPLC) was used to analyze the chemical contents of EtOAc extract. The HPLC chromatogram of EtOAc extract (Figure 4) revealed gallic acid as the most abundant compound with the amount of 15.6% ± 0.06% *w*/*w*. Moreover, corilagin and ellagic acid were only detected in small amounts of 2.2% ± 0.03% *w*/*w* and 2.6% ± 0.02% *w*/*w*, respectively. Apart from our previous study^10^, the chemical compositions of black *D. longan* seeds have rarely been investigated. This study's findings, which identified gallic acid as the predominant component of black *D. longan* seeds extracted using ethyl acetate, were consistent with our earlier research on *D. longan* seed ethanolic extract^10^. Corilagin and gallic acid were the compounds responsible for MMP-1-inhibitory activity of black *D. longan* seed extracts as they exerted potent MMP-1 inhibition with a comparable IC_50_ value to oleanolic acid (Table 1), which is a well-known MMP-1 inhibitor. On the other hand, gallic acid and ellagic acid could be the bioactive compounds responsible for hyaluronidase inhibition since they possessed potent OAEs of 0.52 ± 0.01 and 0.53 ± 0.01 g of oleanolic acid per 1 g sample, respectively (Figure 2). Therefore, gallic acid, corilagin, and ellagic acid are suggested for use as bioactive markers in further quantitative analysis of black *D. longan* seed extracts.

### Lamellar liquid crystals (LLCs)

Various factors affecting the LLC formation, including types and amounts of surfactants, thickening agents, or oils, were investigated in the current study.

#### Effect of surfactant types and amounts

**Figure 5:**
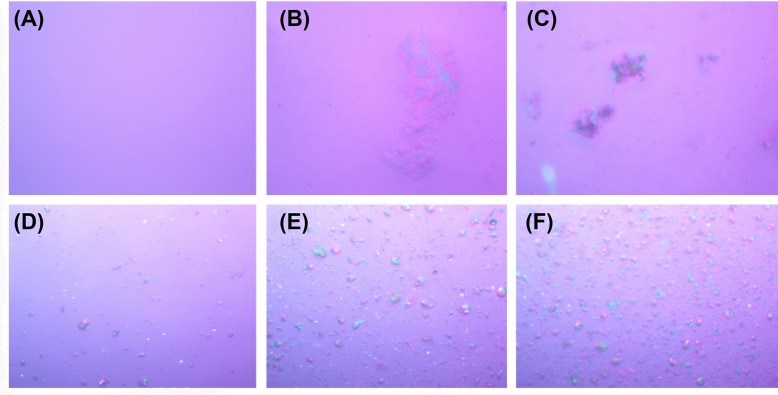
Liquid crystal formation under polarized light optical microscope (magnification 10×) of formulations containing LC with the concentration of 1% *w*/*w* (**A**), 2% *w*/*w* (**B**), and 3% *w*/*w* (**C**), and LCM with the concentration of 1% *w*/*w* (**D**), 2% *w*/*w* (**E**), and 3% *w*/*w* (**F**).

LLCs were developed using two different emulsifying systems, C20–22 alkyl phosphate and C20–22 fatty alcohol (Runemul^TM^ Favor LC:LC), and sorbitan stearate and sucrose cocoate (Liquid Crystal Cream Maker: LCM). The physical appearances of LLCs slightly differed depending on the concentrations of LC and LCM in the formulations. Most formulations were opaque homogeneous semisolid, except for the formulation composed of 1% *w*/*w* surfactant. Therefore, it could be concluded that 1% *w*/*w* LC or LCM was not enough to produce a stable LLC formulation. The minimum amount of surfactant (LC and LCM) required for LLC preparation was 2% *w*/*w*. LCM could produce more pronounced birefringence under the polarizing light microscope than LC, as shown in Figure 5. Since birefringence represents crystallinity and is used to identify LLC formations^31^, LCM is suggested as the effective surfactant for the LLC formation. Furthermore, a higher concentration of each surfactant enhanced the crystallinity of the formulations. Although the viscosity of each formulation (Table 2) revealed that a higher amount of the surfactant led to higher viscosity, the viscosity of the formulations was not related to their crystallinity. LC could produce a formulation with higher viscosity than LCM at the same concentration. A previous study reported that the rheological properties of LLC formulation constituted their crystallinity and complexity^32^. The likely explanation for the results in this study might be due to the difference in the formulation type, whereby LCM produced LLCs but LC produced conventional emulsions. However, all formulations were stable after heating–cooling cycles because their viscosity and birefringence remained unchanged. In brief, 2% *w*/*w* LCM was selected for further study because it could produce LLC formulations with stable crystallinity, and only the minimum concentration was used.

**Table 2:** Viscosity of lamellar liquid crystals (LLCs) using various surfactant types and amounts before and after eight heating–cooling cycles to determine stability.

Concentration (% w/w)	Viscosity (mPa s)			
	LC		LCM	
	Before	After	Before	After
2	0.10 ± 0.04	0.14 ± 0.02	0.01 ± 0.00	0.01 ± 0.00
3	0.18 ± 0.02	0.22 ± 0.03	0.13 ± 0.02	0.13 ± 0.02

LC, C20–22 alkyl phosphate and C20–22 fatty alcohol (Runemul^TM^ Favor LC); LCM, sorbitan stearate and sucrose cocoate (Liquid Crystal Cream Maker).

#### Effect of thickening agent types and amounts

**Figure 6:**
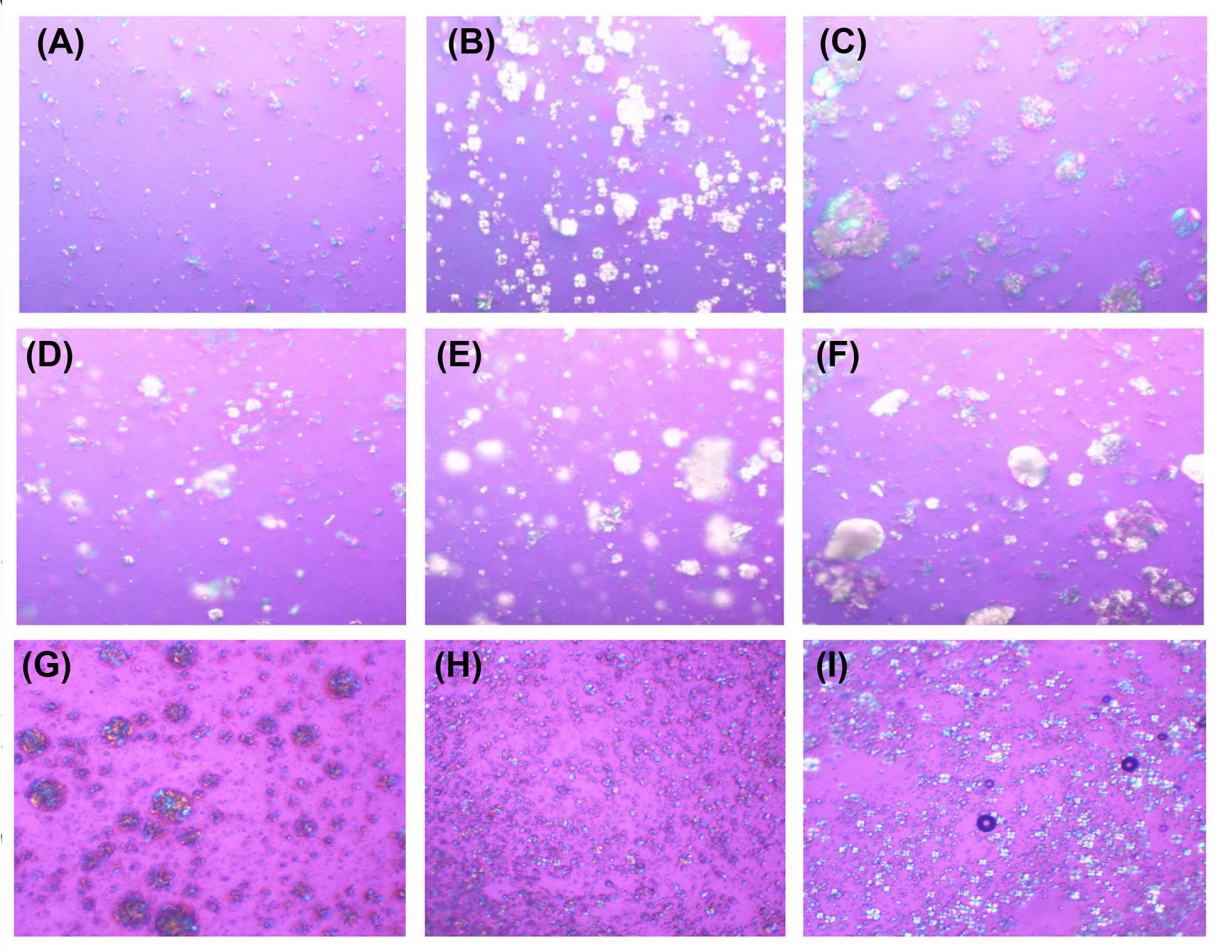
Liquid crystal formation under polarized light optical microscope (magnification 10×) of formulation containing xanthan gum with the concentration of 0.1% *w*/*w* (**A**), 0.5% *w*/*w* (**B**), and 1% *w*/*w* (**C**), Carbopol® U21 with the concentration of 0.1% *w*/*w* (**D**), 0.5% *w*/*w* (**E**), and 1% *w*/*w* (**F**), and sodium carboxymethylcellulose (SCMC) with the concentration of 0.5% *w*/*w* (**G**), 1% *w*/*w* (**H**), and 2% *w*/*w* (**I**).

Since the incorporation of gelator molecules or so-called thickening agents into the lyotropic system resulted in a higher translational order for the gelled lamellar phases in comparison with gelator-free systems^33^, a variety of thickening agents, including xanthan gum, Carbopol^®^ U21, and sodium carboxymethylcellulose (SCMC), were added to the LLC formulations of 2% *w*/*w* LCM. Each formulation had a homogeneous opaque semisolid appearance with birefringence detected under the polarized light optical microscope (Figure 6). Higher concentrations of thickening agents led to more pronounced birefringence, which was well related to their viscosity (Table 3). Xanthan gum and Carbopol^®^ U21 presented the same pattern of birefringence, whereas SCMC presented a small and widely dispersed pattern of birefringence. At the same concentration, the formulation of Carbopol^®^ U21 had a lower viscosity than that of xanthan gum. However, a large amount of SCMC was required for the same viscosity as xanthan gum. Regarding the natural color of xanthan gum, the formulation containing xanthan gum was pale yellow except at the concentration of 0.1% *w*/*w*, at which point the formulation was white. The xanthan gum LLC became a more intense yellow color upon increasing the xanthan gum concentration. In contrast, the formulation composed of Carbopol^®^ U21 was white, while that composed of SCMC was pale yellow without a color change upon increasing the concentration of the thickening agent.

**Table 3:** Viscosity of lamellar liquid crystals (LLCs) using various thickening agent types and amounts before and after eight heating–cooling cycles to test stability.

Thickening agent	Concentration (% *w*/*w*)	Viscosity (mPa s)	
		Before	After
–	–	0.01 ± 0.00	0.01 ± 0.00
Xanthan gum	0.1	0.25 ± 0.01	0.40 ± 0.03**
	0.5	1.36 ± 0.22	1.57 ± 0.26
	1	1.93 ± 0.50	2.44 ± 0.08
Carbopol® U21	0.1	0.05 ± 0.01	0.03 ± 0.00
	0.5	0.10 ± 0.01	0.08 ± 0.01
	1	0.21 ± 0.01	0.20 ± 0.01
SCMC	0.5	0.16 ± 0.01	0.21 ± 0.01**
	1	0.43 ± 0.01	0.42 ± 0.08
	2	2.26 ± 0.20	1.67 ± 0.33

NOTE: LC = C20–22 alkyl phosphate and C20–22 fatty alcohol (Runemul^TM^ Favor LC); LCM = sorbitan stearate and sucrose cocoate (Liquid Crystal Cream Maker). Asterisks (**) denote a significant difference in viscosity before and after the heating–cooling stability test (*p* < 0.01).

All LLC formulations remained stable after heating–cooling cycles in terms of external appearance and crystallinity. However, the viscosity of LLCs containing a low concentration of xanthan gum (0.1% *w*/*w*) and SCMC (0.5% *w*/*w*) was significantly enhanced after the stability test. The likely explanation might be due to the full hydration of the thickening agents after long-term storage. Xanthan gum, an exocellular biopolysaccharide produced by *Xanthomonas campestris*, has been reported to produce a gel-like structure at concentrations of 0.5% and above, while a gel-like structure is absent at 0.25% and below^34^. The results are in good agreement with the present study since the birefringence of the LLC formulation with 0.1% *w*/*w* xanthan gum (Fig. 6A) was the same as that of the LLC formulation without any thickening agent (Fig. 5E). However, the addition of xanthan gum to the LLC formulation resulted in higher viscosity since even a tiny quantity of polysaccharide can considerably enhance the viscosity of either water or the emulsion system^35^. However, the microstructure of xanthan gum is thermally unstable, i.e., sensitive to temperature changes with poor temperature resistance, because heating resulted in a progressive degradation of its network structure^36^. On the other hand, the viscosity of LLCs with a low concentration of SCMC, a semisynthetic anionic cellulose ether, increased dramatically after the stability test. The likely explanation might be due to the high sorption and great hygroscopicity of cellulose derivatives, as well as the structural changes in the native state upon changing temperature^37,38^. In addition to the unstable viscosity after heating–cooling cycles of LLCs from SCMC and xanthan gum, the yellow color was a limitation of using SCMC, while the sticky feeling after application was a limitation of using xanthan gum. Therefore, Carbopol® U21 is suggested as a suitable thickening agent in the LLC formulation for further studies.

#### Effect of various oil types

**Figure 7:**
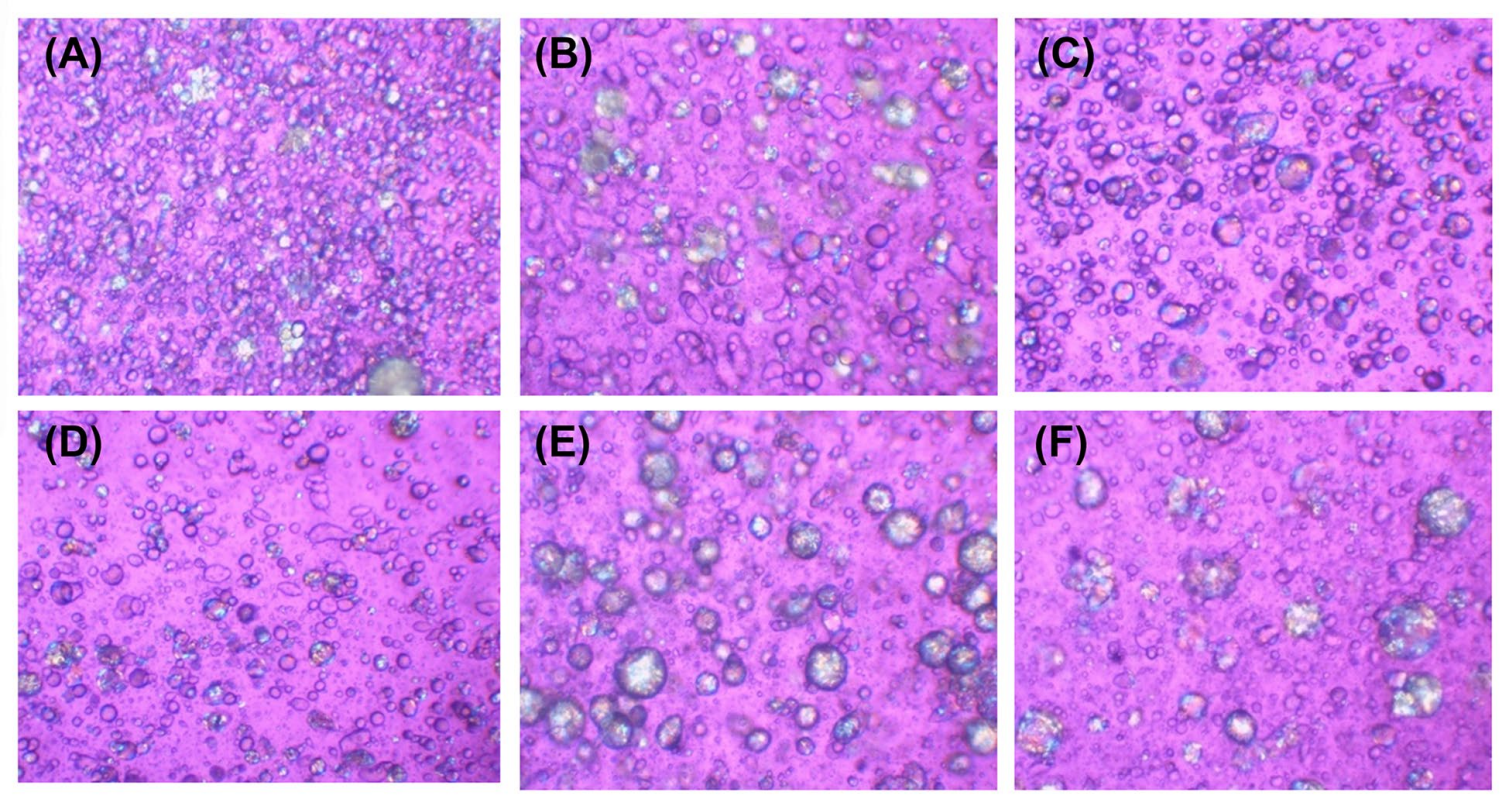
Liquid crystal formation under polarized light optical microscope (magnification 10×) of formulation containing *Simmondsia chinensis* (jojoba) oil (**A**), *Prunus dulcis* (almond) oil (**B**), *Perilla ocymoides* (perilla) seed oil (**C**), *Camellia oleifera* (tea) seed oil (**D**), mineral oil (**E**), and a mixture of neopentyl glycol diheptanoate and isododecane (Lexfeel® D5 oil) (**F**).

Various lipophilic components have been successfully used for LLC development, such as medium-chain triglycerides^39^, alkanes^40^, and edible oils^41^. A variety of oils, both synthesized and natural, were used in the liquid crystal ​development, including *S. chinensis* oil, *P. dulcis* oil, *P. ocymoides* seed oil, *C. oleifera* seed oil, mineral oil, and Lexfeel^®^ D5 oil. The addition of oils to the formulation resulted in increased crystallinity (Figure 7) and viscosity (Table 4). Different oils could generate LLC formulations with different texture, odor, and color. However, the same concentration (5% *w*/*w*) of each oil had no effect on the viscosity. Lexfeel^®^ D5 oil, a mixture of synthesized neopentyl glycol diheptanoate and isododecane, was selected for further study since it produced LLCs that were light, dry, odorless, and colorless.

**Table 4:** Viscosity of lamellar liquid crystals (LLCs) using various oil types before and after eight heating–cooling cycles to test stability.

Oil	Concentration (% *w*/*w*)	Viscosity (mPa s)	
		Before	After
–	–	0.10 ± 0.01	0.08 ± 0.01
Jojoba oil	5	1.94 ± 0.50	2.66 ± 0.40
Almond oil	5	1.49 ± 0.14	1.53 ± 0.22
Perilla seed oil	5	1.74 ± 0.23	1.99 ± 0.08
Tea seed oil	5	1.31 ± 0.53	1.69 ± 0.15
Mineral oil	5	1.75 ± 0.05	2.02 ± 0.13*
Lexfeel® D5 oil	5	1.83 ± 0.22	1.82 ± 0.08

Asterisks (*) denote a significant difference in viscosity before and after the heating–cooling stability test (*p < 0.05).

### Lamellar liquid crystals containing D. longan seed extracts

**Figure 8:**
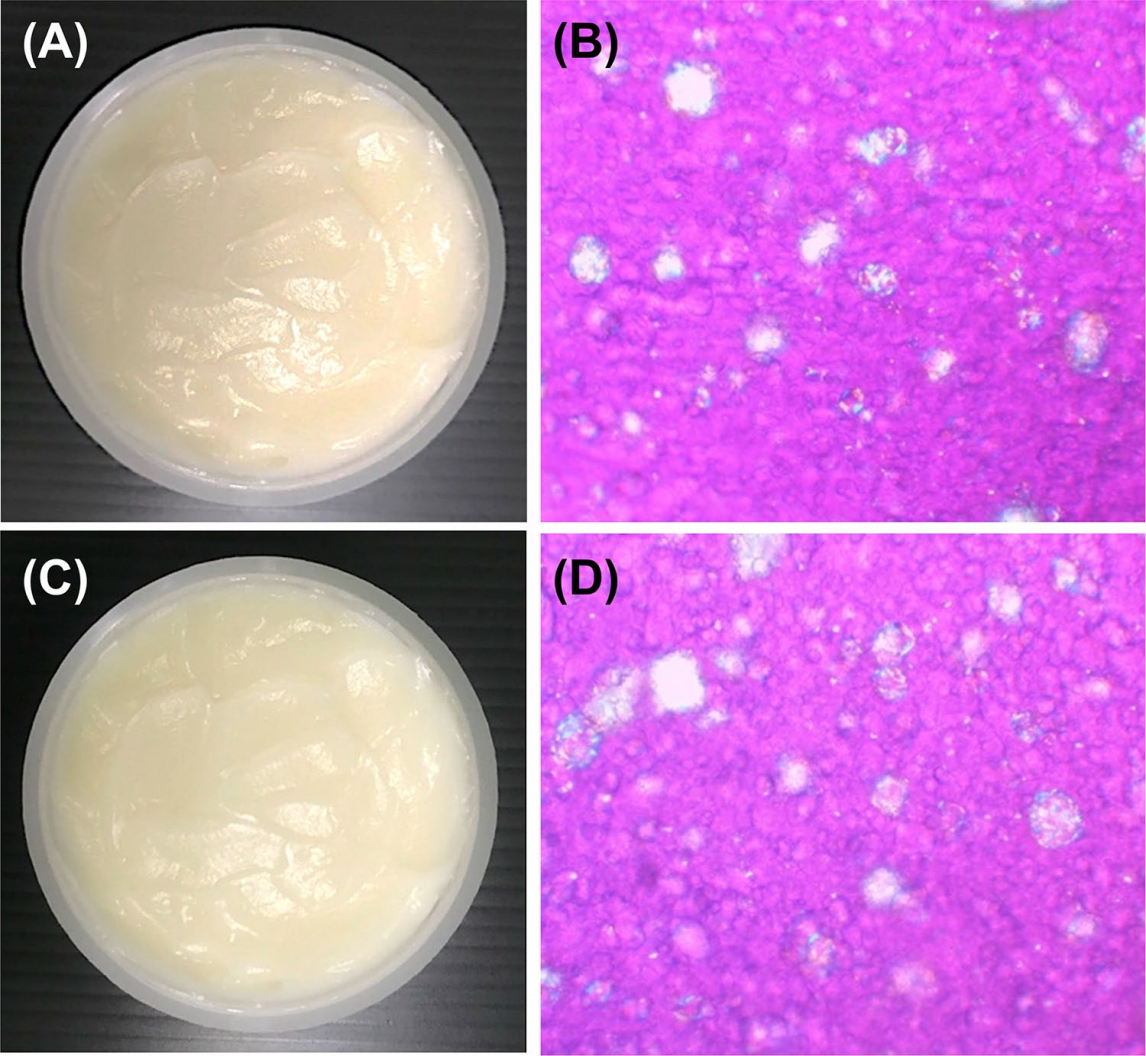
Physical appearance and photo taken under polarized light optical microscope (magnification 10×) of lamellar liquid crystal formulation containing *D. longan* seed ethyl acetate extract (LLC-EtOAc) before (**A**) and after eight heating–cooling cycles to test stability (**B**).

Considering the most potent MMP-1-inhibitory activity equivalent to oleanolic acid, corilagin, and gallic acid, as well as the most potent hyaluronidase-inhibitory activity equivalent to gallic acid and ellagic acid, EtOAc extract was selected as a natural active cosmeceutical ingredient for anti-ageing. However, to achieve inhibition against MMP-1 and hyaluronidase, the active compounds must be delivered to the dermis layer, which is the target site of action. An LLC, containing 5% *w*/*w* Lexfeel^®^ D5 oil, 0.5% *w*/*w* Carbopol^®^ U21, 2% *w*/*w* LCM, and 92.5% *w*/*w* DI water, was developed for delivering EtOAc extract into the skin layer. As only a proportion of EtOAc extract in the LLC formulation can be delivered to the target site (dermis layer), the concentration of EtOAc extract employed in the formulation was made as high as practicable. The concentration of EtOAc extract in the LLC formulation was 0.5% *w*/*w*, with 250 times the IC_50_ value against MMP-1. At this concentration, EtOAc extract caused no irritation. Furthermore, there was no problem with the dissolution of EtOAc extract in the LLC formulation. The LLC formulation containing *D. longan* seed extract (LLC-EtOAc) was a pale-yellow semisolid (Figure 8A) with characteristic odor. Birefringence (Figure 8B) indicated the liquid crystal nature of the formulation. After incorporation of EtOAc extract in the LLC formulation, viscosity increased from 1.83 ± 0.22 to 2.07 ± 0.06 mPa·s (*p* < 0.05). The formulation exhibited good stability after heating–cooling cycles in terms of external appearance, crystallinity, viscosity, and pH (5.5). The external appearance of the formulation remained unchanged in texture, odor, and color (Figure 8C). Furthermore, the birefringence was also the same (Figure 8D). The viscosity of the LLC-EtOAc after the accelerated stability study was 2.22 ± 0.08 mPa·s, which was not different from the initial viscosity (*p* > 0.05). These findings highlight LLC-EtOAc as a stable formulation. However, further analysis of the bioactive compounds in the formulation using the stability test is proposed.

## Conclusions

Black *D. longan* seed extracts, particularly EtOAc extract, have the potential to be used as bioactive cosmetic ingredients. According to the most potent inhibitory activities toward MMP-1 and hyaluronidase, EtOAc extract is of interest to be further investigated for its phytochemical constituents and safety profile. Gallic acid was identified as the most abundant compound (15.6% ± 0.06% w/w) responsible for the biological activities of EtOAc extract. The inhibition of both MMP-1 and hyaluronidase by EtOAc extract was equivalent to gallic acid. The irritation effect of black *D. longan* seed extracts, investigated by the HET-CAM test, revealed their safety as none of the extracts induced any irritation signs on the CAM. Considering its potential anti-skin ageing properties with no irritation, EtOAc extract was incorporated into an LLC, and it is recommended as a potential cosmeceutical formulation for anti-skin wrinkling via MMP-1- and hyaluronidase-inhibitory activities. The findings from this research encourage the utilization of black *D. longan* seeds, which would not only reduce the waste product but also enhance the economic value of *D. longan* by processing it into a natural anti-ageing cosmetic ingredient and finally into cosmetic products. However, prior to launching skincare products containing black *D. longan* seed extracts, further studies on human volunteers evaluating the safety and efficacy of the LLC containing EtOAc extract are suggested. The bioactive compounds in the formulation should also be investigated, along with a stability study to determine the shelf-life of the product.

## Materials and methods

### Chemical materials

Gallic acid, corilagin, ellagic acid, bovine testicular hyaluronidase (E.C.3.2.1.3.5), *Clostridium histolyticum* collagenase (ChC–EC.3.4.23.3, MMP‐1), hyaluronic acid, *N*-[3-(2-furyl) acryloyl]-Leu-Gly-Pro-Ala (FALGPA), disodium phosphate (Na_2_HPO_4_), sodium dihydrogen phosphate (NaH_2_PO_4_), formic acid, hydrochloric acid (HCl), and triethanolamine were purchased in analytical grade from Sigma-Aldrich (St. Louis, MO, USA). Bovine serum albumin (BSA) was purchased from Gibco™ (Thermo Fisher Scientific, Waltham, MA, USA). Acetic acid, ethanol, and dimethyl sulfoxide (DMSO) were purchased in analytical grade from Labscan, Ltd. (Dublin, Ireland). Acetonitrile was purchased in HPLC grade from Labscan, Ltd. (Dublin, Ireland). The mixture of sucrose stearate, cetearyl glucoside, and cetyl alcohol (Runemul^TM^ Favor LC) was purchased in cosmetic grade from Chem Sources Ltd., Part (Bangkok, Thaoland). The mixture of sorbitan stearate and sucrose cocoate (Liquid Crystal Cream Maker) was purchased in cosmetic grade from Chanjao Longevity Co., Ltd. (Bangkok, Thailand). Acrylates/C10–30 alkyl acrylate cross-polymer (Carbopol^®^ Ultrez 21), xanthan gum, sodium carboxymethylcellulose (SCMC), *Simmondsia chinensis* (jojoba) seed oil, *Prunus amygdalus* Dulcis. (sweet almond) oil, *Perilla ocymoides* seed oil, *Camellia oleifera* (tea) seed oil, the mixture of neopentyl glycol diheptanoate and isododecane (Lexfeel^®^ D5 oil), and mineral oil were purchased from NamSiang Co., Ltd. (Chiang Mai, Thailand).

### Plant material and black D. longan preparation

The fruits of *D. longan* (cultivar Edor), harvested in accordance with WHO Guidelines on Good Agricultural and Collection Practices (GACP) for Medicinal Plants^42^, were from Chiang Mai, Thailand. Black *D. longan* was prepared by a thermal and ageing process according to the previous study of Hong-In et al. (2021)^6^. Briefly, after incubating the whole fruit of dried *D. longan* for 20 days at a controlled temperature of 70 °C and 75% relative humidity, black *D. longan* was developed^6^. The pericarp and aril of black *D. longan* fruit were removed. The seeds of black *D. longan* were collected and ground into fine powder, which was kept in a well-closed container at ambient temperature until further use.

### Preparation of black D. longan seed extracts

The powder of black *D. longan* seeds was macerated in three different solvents, petroleum ether, ethyl acetate, and 95% *v*/*v* ethanol, for 24 h at room temperature. The weight ratio of black *D. longan* seed powder and solvent was 1:3. Each extraction was repeated for three cycles. After that, the extracting solvent was filtered and removed using a rotary evaporator (Buchi Labortechnik GmbH, Essen, Germany). All extracts were stored in well-closed containers in the refrigerator (4–6 ºC) until further use.

### MMP-1-inhibitory activity determination of black D. longan seed extracts

The MMP-1-inhibitory activity of each *D. longan* seed extract was investigated by spectrophotometric methods^43,44^. Various concentrations of the samples, ranging from 0.0125 to 0.8 mg/ml, were investigated for their MMP-1-inhibitory activities. Briefly, 0.5 units/mL of MMP-1 from *Clostridium histolyticum* was mixed with the test solution, followed by a substrate (FALGPA). Immediately, the whole mixture was kinetically measured for the absorbance at 340 nm for 20 min using a DTX880 multimode detector (Beckman Coulter, Indianapolis, IN, USA). The inhibitory activity of each sample was calculated using the following equation: % inhibition = (1 − A/B) × 100, where A is the absorbance of the mixture with the test solution and B is the absorbance of the mixture without the test solution. Oleanolic acid was applied as a positive control. The experiment was carried out three times.

### Hyaluronidase-inhibitory activity determination of black D. longan seed extracts

The hyaluronidase inhibitory activity of each *D. longan* seed extract was investigated by spectrophotometric methods^43,44^. The sample concentration of 0.1 mg/mL was investigated for its hyaluronidase-inhibitory activity. Prior to the experiment, the enzyme activity of hyaluronidase was assessed, and a level of more than 90% enzyme activity was used for further anti-hyaluronidase activity determination. Briefly, 15 units of bovine testis-derived hyaluronidase was mixed with the test solution. After incubation at 37 °C for 10 min, hyaluronic acid was added and incubated for another 45 min. The whole mixture was measured for the absorbance at 600 nm using a DTX880 multimode detector (Beckman Coulter, Indianapolis, IN, USA). The inhibitory activity of each sample was reported as oleanolic acid equivalent on hyaluronidase inhibition. The experiment was carried out three times.

### Irritation test of black D. longan seed extracts by hen’s egg test chorioallantoic membrane (HET-CAM) assay

The irritation of black *D. longan* seed extracts was evaluated by the HET-CAM test^29^. The experiment is a well-known and trustworthy irritation study. Because the age of the animal's embryo was less than half of the incubation period, ethical approval was not required. The fertilized hen eggs were incubated for 7 days at 37.5 ± 0.5° C with 55% ± 7% relative humidity. After opening the air chamber above the eggshell using a rotating cutting blade, the inner membrane was gradually removed. Then, 30 μL of 5 mg/ml sample solutions were dropped onto the chorioallantoic membrane (CAM). The irritation signs on CAM were immediately observed under a stereomicroscope (Olympus, Tokyo, Japan) and continuously observed for 5 min. The initiation time of the signs of irritation was recorded and used for the irritation index score (IS) calculation. The equation for IS calculation was as follows: IS = [(301 − t(h))/300 × 5] + [(301 − t(l))/300 × 7] + [(301 − t(c))/300 × 9], where t(h) is the first time vascular hemorrhage was initiated, t(l) is the first time vascular lysis was initiated, and t(c) is the first time vascular coagulation was initiated. The irritation index score (IS) was classified as follows: 0.0–0.9 no irritation, 1.0–4.9 slight irritation, 5.0–8.9 moderate irritation, and 9.0–21.0 severe irritation. The signs of irritation on CAM were observed again after 60 min for long-term irritation. The aqueous solution of 1% *w*/*v* SLS was applied as a positive control, and normal saline solution (0.9% *w*/*v* NaCl) was applied as a negative control. The experiment was carried out twice.

### Determination of phytochemical constituents of black D. longan seed extracts using high-performance liquid chromatography (HPLC)

The amounts of gallica acid, corilagin, and ellagic acid were evaluated using an HP 1100 chromatographic system (Hewlett-Packard, Waldbronn, Germany)^6^. A 0.45 mm millipore filter, type GV (Millipore, Bedford, MA) was used for the filtration of both the test solution and the mobile phase. A gradient mobile phase system, consisting of 0.05% formic acid in acetonitrile (A) and 0.05% formic acid aqueous solution (B), was programmed for gradient elution through a Eurospher II 100-5 C18 column (250 × 4.6 mm, i.d. 5 µm, Knauer, Berlin, Germany) as follows: 10% A (0–8 min), 20% A (8–28 min), 30% A (28–30 min), and 10% A (30–35 min). Each *D. longan* seed extract was dissolved in acetonitrile, and the sample solution was eluted at a flow rate of 1.0 mL/min. The content of ellagic acid was calculated using the area under the curve of each peak detected by a UV detector set at 280 nm.

### Development of lamellar liquid crystals (LLCs)

LLCs were developed using C20–22 alkyl phosphate and C20–22 fatty alcohol (Runemul^TM^ Favor LC) or sorbitan stearate and sucrose cocoate (Liquid Crystal Cream Maker). Various factors affecting liquid crystal systems were investigated, including the type and concentration of surfactant, type and amount of thickening agent, and type and amount of oil.

### Effects of surfactant types and concentrations

To investigate the effects of types and concentrations of surfactants, various formulations of LLC were developed, with the components shown in Table 5. Each liquid crystal system was prepared using the conventional beaker method. Concisely, an aqueous phase (deionized water and moisturizing agents) was mixed and heated until 75 °C in a glass beaker. Simultaneously, an oil phase was heated until 70 °C in another glass beaker. The oil phase was gradually introduced into an aqueous phase and stirred using a stirring rod while cooling to room temperature. Each formulation was kept in a well-closed container at room temperature until the next experiment.

**Table 5:** Formulations of LLCs using different types and amounts of surfactants.

Ingredients	Concentration (% *w*/*w*)					
	1	2	3	4	5	6
LC	1	2	3	–	–	–
LCM	–	–	–	1	2	3
Triethanolamine	0.15	0.30	0.45	–	–	–
DI water q.s	100	100	100	100	100	100

LC: Runemul^TM^ Favor LC (sucrose stearate, cetearyl glucoside, and cetyl alcohol); LCM: Liquid Crystal Cream Maker (sorbitan stearate and sucrose cocoate). Formulations 1–3 used LC at concentrations ranging from 1 to 3% *w*/*w*, while Formulations 4–6 used LCM at concentrations ranging from 1 to 3% *w*/*w.*

### Effects of thickening agent types and concentrations

To investigate the effects of types and concentrations of thickening agents, various formulations of LLC were developed, with the components shown in Table 6. Each thickening agent was previously dispersed in an aqueous phase. Then, the gelled aqueous phase was heated and used for the LLC preparation using the beaker method as described above. When using Carbopol^®^ Ultrez 21 as the thickening agent, triethanolamine was used as a neutralizing agent. The pH of the formulation was adjusted by adding triethanolamine in the final step of preparation. Each formulation was kept in a well-closed container at room temperature until the next experiment.

**Table 6:** Formulations of LLCs using different types and concentrations of thickening agents.

Ingredients	Concentration (% *w*/*w*)								
	7	8	9	10	11	12	13	14	15
LCM	2	2	2	2	2	2	2	2	2
Xanthan gum	0.1	0.5	1	–	–	–	–	–	–
Carbopol® Ultrez 21	–	–	–	0.1	0.5	1	–	–	–
SCMC	–	–	–	–	–	–	0.5	1	2
Triethanolamine	–	–	–	q.s	q.s	q.s	–	–	–
DI water q.s	100	100	100	100	100	100	100	100	100

LCM: Liquid Crystal Cream Maker (sorbitan stearate and sucrose cocoate); Carbopol® Ultrez 21: acrylates/C10–30 alkyl acrylate cross-polymer; SCMC: sodium carboxymethylcellulose. Formulations 7–9 used xanthan gum at concentrations ranging from 0.1 to 1% *w*/*w*, Formulations 10–12 used Carbopol® Ultrez 21 at concentrations ranging from 0.1 to 1% *w*/*w*, and Formulations 13–15 used SCMC at concentrations ranging from 0.1 to 1% *w*/*w.*

### Effects of oil phase

To investigate the effects of oil type, various formulations of LLC were developed, with the components shown in Table 7. Each liquid crystal system was prepared using the conventional beaker method as previously described. Each formulation was kept in a well-closed container at room temperature until the next experiment.

**Table 7:** Formulations of LLCs using different types of oils.

Ingredients	Concentration (% *w*/*w*)					
	16	17	18	19	20	21
LCM	2	2	2	2	2	2
Carbopol® Ultrez 21	0.5	0.5	0.5	0.5	0.5	0.5
Jojoba oil	5	–	–	–	–	–
Almond oil	–	5	–	–	–	–
Perilla oil	–	–	5	–	–	–
Camelia tea oil	–	–	–	5	–	–
Mineral oil	–	–	–	–	5	–
Lexfeel® D5 oil	–	–	–	–	–	5
Triethanolamine	q.s	q.s	q.s	q.s	q.s	q.s
DI water q.s	100	100	100	100	100	100

LCM: Liquid Crystal Cream Maker (sorbitan stearate and sucrose cocoate); Carbopol® Ultrez 21: acrylates/C10–30 alkyl acrylate cross-polymer; Lexfeel® D5 oil: neopentyl glycol diheptanoate and isododecane. Formulations 16–21 used different oil types: jojoba oil, almond oil, perilla oil, camelia tea oil, mineral oil, and Lexfeel® D5 oil, respectively.

### Effects of oil concentration

To investigate the effects of oil concentration, various formulations of LLC were developed, with the components shown in Table 8. Each liquid crystal system was prepared using the conventional beaker method as previously described. Each formulation was kept in a well-closed container at room temperature until the next experiment.

**Table 8:** Formulations of LLCs using different concentrations of Lexfeel® D5 oil.

Ingredients	Concentration (% *w*/*w*)		
	22	21	23
LCM	2	2	2
Carbopol® Ultrez 21	0.5	0.5	0.5
Lexfeel® D5 oil	1	5	10
Triethanolamine	q.s	q.s	q.s
DI water q.s	100	100	100

LCM: Liquid Crystal Cream Maker (sorbitan stearate and sucrose cocoate); Carbopol® Ultrez 21: acrylates/C10–30 alkyl acrylate cross-polymer; Lexfeel® D5 oil: neopentyl glycol diheptanoate and isododecane. Formulations 21–23 used Lexfeel® D5 oil at concentrations ranging from 1 to 10% *w*/*w.*

### Characterization of LLC formulations

Liquid crystals were characterized for external appearance by organoleptic inspections. The pH was measured using universal pH paper. Viscosity was measured using a Brookfield R/S rheometer (Brookfield, Middleboro, MA, USA) at room temperature. A Motic BA310 POL Trinocular polarizing microscope (Carlsbad, California, USA) was used in order to investigate the liquid crystal characteristics of each formulation.

### Stability test

The stability of each LLC formulation was determined after eight heating–cooling cycles. In each cycle, the LLCs were kept at 45 °C for 24 h and then at 4 °C for 24 h. After that, the LLCs were investigated for external appearance, pH, viscosity, and liquid crystal characteristics.

### Development of LLC containing D. longan seed extracts

The *D. longan* seed extract, which exhibited the highest anti-skin wrinkling activity with no irritation effect, was selected for use as an active cosmetic or cosmeceutical ingredient in the LLC formulation. On the other hand, the LLC formulation, which exhibited good characteristics and stability after the heating–cooling test, was selected for the incorporation of selected *D. longan* seed extract. The formulation of the LLC containing the black *D. longan* seed extract is shown in Table 9. In the preparation process of the LLC containing *D. longan* seed extracts, *D. longan* seed extract was finally added to the preformulated LLC and mixed thoroughly. The LLC formulation containing *D. longan* seed extract was then characterized and tested for their stability via eight heating–cooling cycles.

**Table 9:** Formulation of LLC containing black *D. longan* seed extracts.

Ingredients	Concentration (% *w*/*w*)
EtOAc extract	0.5
LCM	2
Carbopol® Ultrez 21	0.5
Lexfeel® D5 oil	5
Triethanolamine	q.s
DI water q.s	100

EtOAc extract: black *D. longan* seed ethyl acetate extract; LCM: Liquid Crystal Cream Maker (sorbitan stearate and sucrose cocoate); Carbopol® Ultrez 21: acrylates/C10–30 alkyl acrylate cross-polymer; Lexfeel® D5 oil: neopentyl glycol diheptanoate and isododecane.

### Statistical analysis

All values were given as means ± standard deviation. The statistical analysis involved a *t*-test and ANOVA using SPSS software (SPSS Statistics 21.0, IBM Corporations, New York, NY, USA). A value of *p* < 0.05 was accepted as significant.

## Data Availability

The datasets used and analyzed during the current study are available from the corresponding author on reasonable request.
